# Macrophage activation syndrome in rheumatic disease: Clinical characteristics and prognosis of 20 adult patients

**DOI:** 10.1371/journal.pone.0267715

**Published:** 2022-05-06

**Authors:** So Hye Nam, Soo Min Ahn, Ji Seon Oh, Seokchan Hong, Chang-Keun Lee, Bin Yoo, Yong-Gil Kim

**Affiliations:** 1 Division of Rheumatology, Department of Internal Medicine, Eulji University School of Medicine, Uijeongbu Eulji Medical Center, Uijeongbu, Gyeonggi-do, Korea; 2 Division of Rheumatology, Department of Internal Medicine, University of Ulsan College of Medicine, Asan Medical Center, Seoul, Korea; 3 Department of Information Medicine, Asan Medical Center, Seoul, Korea; Meyer Children’s University Hospital - University of Florence, ITALY

## Abstract

**Objectives:**

Macrophage activation syndrome (MAS) is a hyperinflammatory condition that is known to be secondary hemophagocytic lymphohistiocytosis (HLH) in patients with rheumatic disease. The aim of study was to evaluate the clinical manifestations and outcomes in patients with MAS with rheumatic disease.

**Materials and methods:**

We performed a retrospective study of 20 adult patients who were diagnosed with MAS from 2012 to 2020. MAS was classified according to the HLH-2004 criteria. Patients’ information, including clinical features, laboratory findings, and treatment regimens, was collected, and the overall survival rate was estimated by the Kaplan–Meier method.

**Results:**

Twenty patients (18 women, 35.6 ± 18.3 years) who met the HLH-2004 criteria also fulfilled the 2016 EULAR/ACR/PRINTO classification criteria for MAS, and HScore was higher than 169 (mean, 241.1). Fourteen patients with systemic lupus erythematosus and 6 patients with adult-onset Still’s disease were included. All patients were treated initially with corticosteroids, and 16 patients required additional immunosuppressants. The overall survival at 3 and 6 months was 75.2% and 64.3%. In survivors, renal impairment was less common (7.7% versus 71.4%, p = 0.007), the levels of AST (364.0 versus 81.0 IU/L, p = 0.019) and LDH (1346.0 versus 343.0IU/L, p = 0.014), and platelet count (90.0 versus 43.0 × 10^9^/L, p = 0.02) were higher in compared to non-survivors. Nine patients had opportunistic infections, five of whom died during admission.

**Conclusion:**

The mortality of patients with MAS associated with rheumatic disease remains high. Renal impairment, levels of AST and LDH, and platelet count might be associated with prognosis.

## Introduction

Macrophage activation syndrome (MAS), known as secondary hemophagocytic lymphohistiocytosis (HLH), is a phenomenon that is characterized by natural killer (NK) cell and cytotoxic T lymphocyte dysfunction, causing uncontrolled activation of macrophage and production of pro-inflammatory cytokines [1–5]. With the exception of from primary HLH, which is caused by genetic defects, MAS can be triggered by malignancy, infection, and various autoimmune diseases [1–8]. MAS maintains a hyperinflammatory state, and leads to cytokine storm, hemophagocytosis, and multi-organ failure [1–5]. In rheumatic disease, MAS is known to develop most commonly in adult-onset Still’s disease (AOSD) and systemic lupus erythematosus (SLE); however, the incidence of MAS related to rheumatic disease is rare [2–13]. Recently, several studies suggested that the prevalence of MAS in rheumatic disease may be under recognized, as the symptoms of MAS are quite similar to those of many active autoimmune diseases and severe sepsis [2–4, 10–12]. Although previous studies have reported that MAS is associated with rheumatic disease, most of them were included as a minority of patients in the secondary HLH study, or the triggering factors of MAS were often mixed with infection other than rheumatic disease [7, 10–16]. Therefore, the factors influencing prognosis of MAS in patients with rheumatic disease have not yet been fully identified, and appropriate management is still uncertain.

Here, we investigate the characteristics, mortality, and prognosis of Korean patients with MAS with rheumatic disease.

## Materials and methods

### Study population and data collection

In this retrospective cohort study, we reviewed the results of all bone marrow biopsies and laboratory tests in adult (≥ 18 years) patients with underlying rheumatic disease from January 2012 to June 2020 at a tertiary referral hospital in Seoul. All patients with SLE fulfilled the Systemic Lupus International Collaborating Clinics criteria [17]. AOSD was classified using the preliminary criteria for classification of AOSD proposed by Yamaguchi et al. [18]. The diagnosis of MAS was based on the HLH-2004 criteria from the Histiocyte Society, and was required to meet at least five of the following criteria [19]: (1) fever ≥ 38.5°C; (2) splenomegaly; (3) cytopenia involving in at least two lineages of peripheral blood (neutrophil count < 1.0 × 10^9^/L, hemoglobin < 9 g/dL or platelet < 100 × 10^9^/L); (4) hypertriglyceridemia (≥ 265 mg/dL) and/or hypofibrinogenemia (fibrinogen ≤ 150 mg/dL); (5) hemophagocytosis in the bone marrow, spleen, or lymph nodes; (6) low or absent NK-cell activity; (7) serum ferritin ≥ 500 μg/L; and (8) soluble CD25 (soluble interleukin-2 receptor) ≥ 2400 IU/ml. We also checked the HScore and the European League Against Rheumatism (EULAR)/American College of Rheumatology (ACR)/Pediatric Rheumatology International Trials Organization (PRINTO) criteria for MAS diagnosis [20, 21]. We also checked the preliminary diagnostic criteria for MAS in patients with SLE [22]. Patients with MAS associated with non-rheumatic disease, including prior infection or malignancy, were excluded.

The following data were collected from the patients’ electronic medical records: demographic information, including age and sex; underlying rheumatic diseases such as SLE and AOSD; comorbid medical conditions, including transplantation and malignancy; medications, including corticosteroids and immunosuppressants; and laboratory data such as complete blood cell counts, aspartate aminotransferase (AST), fibrinogen, albumin, triglyceride (TG), lactate dehydrogenase (LDH), total bilirubin, erythrocyte sedimentation rate (ESR), C-reactive protein (CRP), and ferritin. We also collected information of the patients’ clinical conditions at the time of MAS diagnosis, including vital signs and neuropsychiatric symptoms. Underlying disease activity at the time of the MAS diagnosis was evaluated using the Systemic Lupus Erythematosus Disease Activity Index (SLEDAI)-2000 in patients with SLE, and the Pouchot score in patients with AOSD [23, 24].

This study was approved by the Institutional Review Boards of Asan Medical Center (IRB No. 2020–1179). The requirement for informed consent was waived owing to the retrospective nature of the analysis.

### Statistical analyses

Categorical variables are presented as frequencies with percentages (n, %), and Chi-square test and Fisher’s exact tests were used to compare. Continuous variables are expressed as means (standard deviations) or medians (interquartile ranges [IQR]) and were calculated using the Student’s t-test for parametric data or the Mann–Whitney U test for nonparametric data. The Kaplan–Meier method with the long-rank test was used for survival curves. A two-side p-value < 0.05 was considered statistically significant. All statistical analyses were performed using SPSS software (version 21.0; IBM, Armonk, NY).

## Results

### Baseline characteristics of patients with MAS

Twenty adult patients (14 patients with SLE, 6 patients with AOSD) were included in this study. Eighteen (90%) patients were female, and the mean age at MAS was 35.6 ± 18.3 years. The mean duration between diagnosis of rheumatic disease and MAS development was 31.3 ± 56.4 (range, 0–182) months. Of the 20 patients, 10 developed MAS within 1 month of being diagnosed with rheumatic disease (5 with SLE, 5 with AOSD). At diagnosis, patients uniformly presented with fever (≥ 38.5°C) and hyperferritinemia (≥ 500 ng/mL). Thrombocytopenia and anemia were noted in 16 patients, and neutropenia was noted in 6 patients. Cytopenia in two lineages was shown in 15 (75%) patients. Hemophagocytosis was identified in 16 (80%) patients upon bone marrow biopsy. Among them, 12 had SLE and 4 had AOSD. The clinical and laboratory findings of patients according to HLH-2004 criteria are presented in Table 1. All patients met the EULAR/ACR/PRINTO criteria, and all had a HScore higher than 169 (median, 238.5; IQR, 205.8–277.8). We also confirmed that the preliminary diagnostic criteria for MAS were met by all SLE patients.

**Table 1:** Diagnostic criteria fulfilled by the 20 eligible patients in HLH-2004.

	n = 20
Fever (≥ 38.5°C)	20 (100)
Splenomegaly	13 (65)
Bicytopenia (2/3 cell lineage)	15 (75)
Hypertriglyceridemia or hypofibrinogenemia	12 (60)
Hypertriglyceridemia ≥ 265 mg/dL	12 (60)
Hypofibrinogenemioa ≤ 150 mg/dL	4 (20)
Hemophagocytosis	16 (80)
Ferritin ≥ 500 ng/mL	20 (100)
Low/absent NK-cell activity	6 (30)
Soluble CD25 ≥ 2400 U/ml	8 (40)
Hscore* > 169	20 (100)
PRINTO criteria	20 (100)

*HScore calculator (for the percentage probability of secondary HLH) is available at http://saintantoine.aphp.fr/score/. HLH: Hemophagocytic lymphohistiocytosis, NK-cell: Natural killer cell, PRINTO: Pediatric Rheumatology International Trials Organization.

### Clinical differences between patients with SLE and AOSD

There was no significant difference between patients with SLE and AOSD in gender and age. Otherwise, the mean disease duration until MAS diagnosis was shorter in patients with AOSD (44.6 months with SLE vs 0.2 months with AOSD, p = 0.038). Thirteen (65%) and seven (35%) patients had splenomegaly and hepatomegaly, respectively. Neuropsychiatric symptoms were present in six patients, five of whom had SLE. Seizure and encephalopathy were the most common symptoms. Renal impairment occurred in six patients, and five of them needed hemodialysis (4 patients with SLE, 1 patient with AOSD). Elevated LDH was found in 19 patients (95%), liver enzyme in 17 (85%), and hypertriglyceridemia in 12 (60%) (Table 2). There was no significant difference between laboratory findings except for CRP (1.4 mg/dL with SLE vs 6.0mg/dL with AOSD, p = 0.048).

**Table 2:** Baseline characteristics of 20 patients with MAS.

	Total (n = 20)	SLE (n = 14)	AOSD (n = 6)
Sex, female, n (%)	18 (90)	13 (92.9)	5 (83.3)
Age, years, mean ± SD	35.6 ± 18.3	37.4±16.2	39.7±18.8
Disease duration to MAS occurrence^¶^, months, mean ± SD	31.3±56.4	44.6±63.4	0.2±0.4
Clinical features			
Hepatomegaly, n (%)	7 (35)	3 (21.4)	4 (66.7)
Lymphadenopathy, n (%)	14 (70)	10 (71.4)	4 (66.7)
Pneumonitis, n (%)	6 (30)	5 (35.7)	1 (16.7)
Renal impairment^¶¶^, n (%)	6 (30)	5 (35.7)	1 (16.7)
Neuropsychiatric manifestations, n (%)	6 (30)	5 (35.7)	1 (16.7)
Lab findings			
ANC, /uL, median (IQR)	2890 (875, 8285)	2544.5 (590.0, 5512.5)	5420.0 (1605.0, 11877.5)
Hb, g/dL, median (IQR)	8.5 (7.2, 9.0)	8.6 (7.5, 9.1)	7.6 (6.5, 9.3)
PLT, x 10^9^/L, mean ± SD	101.3±95.1	66.6±30.6	182.2±144.0
AST, IU/L, median (IQR)	137.5 (75.8, 293.0)	155.0 (91.5, 564.8)	506.0 (72.8, 919.0)
LDH, IU/L, median (IQR)	899.5 (381.5, 1556.8)	648.5 (358.0, 1729.8)	1221.0 (743.5, 3381.3)
TB, mg/dL, median (IQR)	0.8 (0.5, 2.8)	0.5 (0.3, 3.4)	1.0 (0.8, 2.7)
Albumin, g/dL, mean ± SD	2.4±0.4	2.3±0.4	2.5±0.4
eGFR^$^, mL/min/1.73m^2^, mean ± SD	101.8±40.8	96.4±42.4	114.5±37.6
ESR, mm/hr, median (IQR)	26.5(17.0, 53.0)	30.0 (17.8, 59.5)	21.0 (14.3, 39.0)
CRP^¶^, mg/dL, median (IQR)	2.2 (0.6, 6.3)	1.4 (0.3, 3.4)	6.0 (2.3, 6.6)
Ferritin, ng/mL, median (IQR)	6039.2 (2875.2, 21027.0)	4967.3 (2706.7, 10553.9)	17866.9 (9822.0, 84821.3)
TG, mg/dL, mean ± SD	297.1±134.5	277.9±138.3	309.5±147.2
Fibrinogen, mg/dL, median (IQR)	215.5 (154.8, 272.0)	220.5 (184.8, 285.5)	147.5 (86.3, 249.0)
Hemophagocytosis, n, (%)	16 (80)	12 (85.7)	4 (66.7)
Hscore*, mean ± SD	241.1 ± 42.9	237.9±38.8	248.5±54.7
Underlying disease activity^¥^		20.2±7.9	7.5±1.4

^¶^ p-value < 0.05.

*HScore calculator (for the percentage probability of secondary HLH) is available at http://saintantoine.aphp.fr/score/.

^¶¶^Renal impairment was defined as an abrupt (within 48 hours) >50% decrease in eGFR or the need for renal replacement therapy (dialysis) during MAS treatment.

^$^eGFR is estimated using an equation developed by the Chronic Kidney Disease Epidemiology Collaboration.

^¥^SLEDAI-2K for SLE and Pouchot score for AOSD. MAS: Macrophage activation syndrome, HLH: Hemophagocytic lymphohistiocytosis, SLE: Systemic lupus erythematosus, AOSD: Adult-onset still’s disease, WBC: White blood cell, Hb: Hemoglobin, PLT: Platelet, AST: Aspartate aminotransferase, LDH: Lactate dehydrogenase, TB: Total bilirubin, eGFR, estimated glomerular filtration rate; ESR: Estimated sedimentation rate, CRP: C-reactive protein, TG: Triglyceride. SLEDAI-2K, Systemic Lupus Erythematosus Disease Activity Index-2000

### Treatment and outcome of patients with MAS

**Fig 1:**
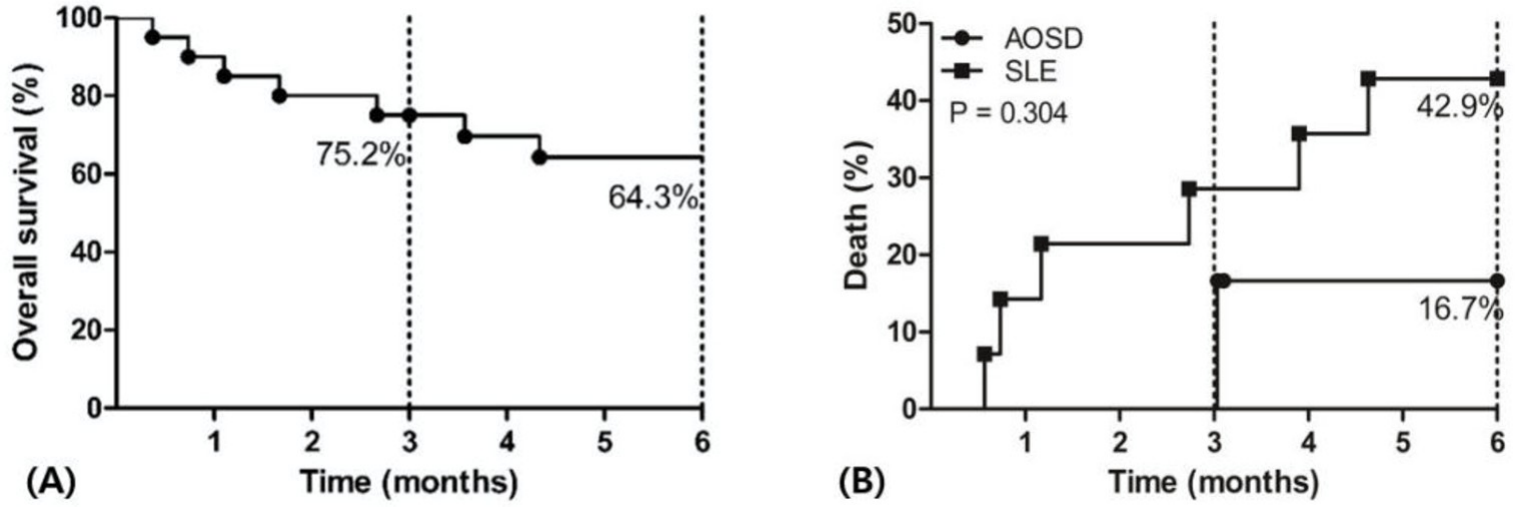
Overall survival (A) and mortality (B) of patients with MAS within 6 months MAS: Macrophage activation syndrome, AOSD: Adult-onset still’s disease, SLE: Systemic lupus erythematosus.

Most of the patients received high dose corticosteroids (≥ prednisolone equivalent 1 mg/kg), and one patient received 0.5 mg/kg. The mean duration from admission to administration of corticosteroids was 2.5 ± 4.7 days (median, 0.5, IQR, 0–2.8). During the management of MAS, ten patients were admitted to the intensive care unit due to increased oxygen demand and multi-organ failure, and two of them died during the first 30-days after admission. Seven patients died after diagnosis of MAS. The overall survival rate was 75.2% and 64.3% at 3 months and 6 months, respectively (Fig 1A). In terms of the types of rheumatic disease, the mortality rate of MAS appeared to be higher in patients with SLE (42.9%) than those with AOSD (16.7%), although there was no significant difference (Fig 1B).

The treatments and outcomes of patients are summarized in Table 3. Sixteen (80%) patients were considered refractory to high dose corticosteroids, and consequently received immunosuppressive agents or intravenous immunoglobulin (IVIG) as an alternative. Tocilizumab was administered in four patients; however, two patients died within 4 and 17 days after infusion, and the others required additional treatment, including rituximab and cyclosporin. Etoposide was used in five patients; three of them, including a patient co-treated with tocilizumab, died after administration of etoposide, and the remaining two patients were discharged alive. Ten patients experienced viral, bacterial, and fungal infection during hospitalization. In one AOSD patient, parvovirus was detected at the time of MAS diagnosis in bone marrow tissue; however, the association with MAS was unclear. Opportunistic infections developed in 9 patients during treatment; three patients had fungal infections after immunosuppressive treatment such as etoposide or cyclosporin (one patient with fungemia and two patients with invasive fungal sinusitis, both requiring surgery and antibiotics).

**Table 3:** Treatments and management characteristics of patients with MAS.

No.	Age/sex	Disease	Disease duration (months)	1^st^ Treatment (corticosteroids)	2^nd^ Treatment	3^rd^ Treatment	Combined infection	Alive/dead
1	19/F	SLE	1	1 mg/kg	IVIG + PP	TCZ, RTX	Bacteremia	Dead
2	20/M	SLE	0	1 mg/kg	-	-	-	Alive
3	20/F	AOSD	1	1 mg/kg	VP16	-	-	Alive
4	22/F	SLE	1	100 mg	IVIG + PP	-	Pneumonia	Dead
5	22/F	AOSD	0	500 mg	IVIG	-	-	Alive
6	23/F	SLE	182	1 mg/kg	-	-	-	Alive
7	23/F	SLE	41	1 mg/kg	-	-	-	Alive
8	30/F	SLE	146	1 mg/kg	IVIG	CsA	-	Alive
9	32/F	SLE	127	1 mg/kg	IVIG + PP	CsA, TCZ	Pneumonia	Alive
10	35/F	AOSD	0	1 mg/kg	CsA	-	Viral infection	Alive
11	37/F	SLE	65	1 mg/kg	CsA, VP16	-	Bacteremia	Alive
12	38/F	SLE	0	1 mg/kg	IVIG + PP	RTX	-	Dead
13	40/F	AOSD	0	0.5 mg/kg	CsA	-	-	Alive
14	43/F	SLE	60	1 mg/kg	IVIG + PP	TCZ, RTX, CsA, VP16, IFX	PCP, Viral infection	Dead
15	49/F	SLE	0	1 mg/kg	CYC	-	Bacteremia	Alive
16	51/F	AOSD	0	1 mg/kg	-	-	-	Alive
17	57/F	SLE	0	1 mg/kg	IVIG + PP	CsA, VP16	Fungal infection	Dead
18	61/F	SLE	2	1 mg/kg	IVIG + PP	TCZ	-	Dead
19	68/F	SLE	2	1 mg/kg	IVIG + PP	CsA	Fungal infection	Alive
20	70/M	AOSD	0	1 mg/kg	IVIG + PP	CsA, VP16	Fungal infection	Dead

SLE: Systemic lupus erythematosus, IVIG: Intravenous immunoglobulin, PP: Plasmapheresis, TCZ: Tocilizumab, RTX: Rituximab, AOSD: Adult-onset still’s disease, VP16: Etoposide, PCP: Pneumocystis pneumonia, CsA: Cyclosporin, IFX: Infliximab

### Clinical features of survivors and non-survivors

As shown in Table 4, we performed further analysis to establish the difference in clinical features according to the outcomes of patients with MAS. Renal impairment was observed more commonly in non-survivors (7.7% versus 71.4%, p = 0.007), otherwise, the levels of AST (364.0 versus 81.0 IU/L, p = 0.019) and LDH (1346.0 versus 343.0IU/L, p = 0.014), and platelet count (90.0 versus 43.0 × 10^9^/L, p = 0.002) were higher in survivors than those in non-survivors. Age, presence of infection, previous use of corticosteroids, and the mean HScore were not significantly different between survivors and non-survivors.

**Table 4:** Comparison of characteristics between survivors and non-survivors in patients with MAS.

	Survivors (n = 13)	Non-survivors (n = 7)	p-value
Sex, female, n (%)	12 (92.3)	6 (85.7)	1.000
Age, years, mean ± SD	34.6 ± 14.5	44.4 ± 19.4	0.215
Disease duration to MAS occurrence, months, median (IQR)	1.0 (0.0, 96.0)	0 (0.0, 1.0)	0.393
Clinical features			
Splenomegaly, n (%)	10 (76.9)	3 (42.9)	0.174
Hepatomegaly, n (%)	6 (46.2)	1 (14.3)	0.329
Lymphadenopathy, n (%)	10 (76.9)	4 (57.1)	0.613
Pneumonitis, n (%)	3 (23.1)	3 (42.9)	0.613
**Renal impairment**^**¶¶**^**, n (%)**	**1 (7.7)**	**5 (71.4)**	**0.007**
Neuropsychiatric manifestations, n (%)	5 (38.5)	1 (14.3)	0.354
Lab findings			
ANC, /uL, median (IQR)	2880 (685, 6475)	3240 (890, 9260)	0.588
Hb, g/dL, median (IQR)	8.7 (7.6, 9.2)	7.6 (6.4, 8.9)	0.183
**PLT, × 10**^**9**^**/L, median (IQR)**	**90.0 (61.0, 149.0)**	**43.0 (30.0, 74.0)**	**0.002**
** AST, IU/L, median (IQR)**	**364.0 (137.0, 689.0)**	**81.0 (37.0, 157.0)**	**0.019**
** LDH, IU/L, median (IQR)**	**1346.0 (655.5, 1893.5)**	**343.0 (279.0, 733.0)**	**0.014**
TB, mg/dL, median (IQR)	0.8 (0.4, 1.8)	0.8 (0.5, 4.5)	0.699
Albumin, g/dL, median (IQR)	2.4 (2.1, 2.7)	2.2 (1.8, 2.8)	0.588
ESR, mm/hr, median (IQR)	28.0 (17.5, 52.0)	23.0 (6.0, 67.0)	0.588
CRP, mg/dL, median (IQR)	1.6 (0.4, 4.0)	6.1 (2.2, 22.0)	0.056
Ferritin, ng/mL, median (IQR)	16817.0 (2811.5, 48286.0)	5409.7 (2784.1, 6395.3)	0.183
TG, mg/dL, median (IQR)	322.5 (209.5, 436.3)	176.0 (137.0, 403.0)	0.299
Fibrinogen, mg/dL, median (IQR)	214.0 (148.5, 261.0)	217.0 (176.0, 363.0)	1.000
Low/absent NK-cell activity	5 (38.5)	1 (14.3)	0.354
Soluble CD25 ≥ 2400 U/ml	3 (23.1)	5 (71.4)	0.062
Hemophagocytosis, n (%)	11 (84.6)	5 (71.4)	0.587
Hscore*, mean ± SD	251.9 ± 37.8	220.9 ± 47.5	0.135
Combined infection	5 (38.5)	5 (71.4)	0.350
Previous long-term steroid use, n (%)	7 (53.8%)	1 (14.3%)	0.158
Rheumatic disease			0.354
SLE	8 (61.5)	6 (85.7)	
AOSD	5 (38.5)	1 (14.3)	

*HScore calculator (for the percentage probability of secondary HLH) is available at http://saintantoine.aphp.fr/score/.

^¶¶^Renal impairment was defined as an abrupt (within 48 hours) >50% decrease in eGFR or the need for renal replacement therapy (dialysis) during MAS treatment. MAS: Macrophage activation syndrome, HLH: Hemophagocytic lymphohistiocytosis, ANC: Absolute neutrophil count, Hb: Hemoglobin, PLT: Platelet, AST: Aspartate aminotransferase, LDH: Lactate dehydrogenase, TB: Total bilirubin, ESR: Estimated sedimentation rate, CRP: C-reactive protein, TG: Triglyceride, SLE: Systemic lupus erythematosus, AOSD: Adult-onset still’s disease, eGFR, estimated glomerular filtration rate

## Discussion

MAS is a life-threatening phenomenon in patients with rheumatic disease, and early recognition of MAS is essential to improve the morbidity and mortality in patients with rheumatic disease [2–6, 10, 15]. In the present study, we reviewed 20 patients with rheumatic disease who experienced MAS. The overall survival rate was 64.3% at 6 months, SLE and AOSD were the major underlying rheumatic diseases, and half of the cases presented with MAS as an initial manifestation.

As there are no diagnostic markers for MAS, it was difficult to identify in patients with rheumatic disease. Several classification criteria or score systems have been used for the identification of MAS, including the HLH-2004 or EULAR/ACR/PRINTO criteria, however, there remain no defined diagnostic criteria [2, 5, 6]. Leukopenia and thrombocytopenia, both of which are included in the HLH criteria, can even be observed in patients with SLE who have stable disease. Otherwise, as leukocytosis is typical manifestation in patients with AOSD, relying solely on these criteria may delay the diagnosis [5, 25]. The EULAR/ACR/PRINTO criteria, which was originally developed for patients with juvenile idiopathic arthritis, has been suggested for use in adult patients with rheumatic disease, considering that soluble IL-2 and NK-cell activity are not routinely accessible in most hospitals [11, 25]. However, it is still relatively easy to meet the diagnostic criteria because at least two of four clinical criteria (platelet count ≤ 181,000 /μL, AST > 48 units/L, triglyceride > 156 mg/dL, and fibrinogen ≤ 360 mg/Dl) need to be satisfied, with ferritin elevation (> 684 ng/dL) [21]. Therefore, if the physician overlooks the exclusion of other causes, sepsis or disease flare can be mistaken for MAS. The HScore was recently proposed to define reactive hemophagocytic syndrome in adults (not validated in pediatric patients), and Fardet et al. proposed that its best cut-off value was 169 with sensitivity and specificity of 93% and 86%, respectively [20]. However, there is controversy surrounding whether the cut-off value of MAS diagnosis in rheumatic disease should be viewed higher than the reference value because variables such as cytopenia and high ferritin level can be seen in patients with SLE or AOSD but without MAS. Moreover, the HScore was validated using reactive HLH with various underlying diseases, with < 10% of patients with rheumatic disease in the population [20]. Considering that it is difficult to measure all categories of HLH-2004, MAS in rheumatic disease might be under detected or misdiagnosed. Therefore, in the present study, we used the HLH-2004 criteria, and also checked EULAR/ACR/PRINTO criteria and HScore for MAS diagnosis. We confirmed that our patients were satisfied with these criteria.

One of the interesting findings of our study was that the value of the HScore was not correlated with prognosis in our patients. In contrast, previous study has suggested that an elevated HScore is related to poor prognosis [26]. However, this previous study populations did not solely consist of patients with rheumatic disease, and the median Hscore (63.0) was lower than that reported in our study (241.1). Because most of the patients in this study scored high on the variables in the HScore, it might be difficult to predict the prognosis in our patients with rheumatic disease. However, due to the limitation of the small study population, further studies are needed to verify whether the prognosis of MAS is predictable using the HScore.

In the present study, the clinical presentations of MAS varied from the musculoskeletal system to the central nervous system (CNS), and the frequency of these manifestations was similar to that reported by other studies [4, 10, 11, 14]. Among them, the CNS manifestations were predominantly found in patients with SLE patients (5 of 6 patients), however, it did not affect the prognosis. Otherwise, renal impairment was significantly more observed in non-survivors. Another factor that differed between survivors and non-survivors was level of AST and LDH. Survivors at 6 months showed high levels of AST and LDH, which are markers of tissue turnover. This can be explained by the concept that high tissue turnover status might respond well to immunosuppressants. For other reasons, the majority of patients with AOSD, which is characterized by high AST and LDH levels, survived (5 of 13, 38.5%). Although, there were many patients with SLE in non-survivors (6 of 7, 85.7%). we could not find significant difference in mortality between patients with SLE and AOSD. However, given the large number of survivors of AOSD patients (Fig 1B), further studies are needed to determine if MAS patients underlying AOSD are likely to show a reduced mortality compared to those with SLE.

In this study, more than half of the patients were refractory to corticosteroids. Despite the advances in anti-inflammatory agents, the treatment outcome of MAS remains poor. We have tried various treatments, including cyclosporin, IL-6R inhibitor (tocilizumab), and chemotoxic agents such as etoposide. In the case of tocilizumab, there is controversy over the effectiveness of MAS treatment [1–3]. Tocilizumab was suggested to prevent a cytokine storm by blocking IL-6R and disrupting the IL-6 signaling pathway, however, because MAS is attributed to various cytokines, including IL-1 and IL-18, inhibition of IL-6 might not be sufficient to prevent MAS [1–3]. In our study, we tried tocilizumab in four patients, however, all of them failed to reach the treatment goal. Recently, the prospective clinical trial of ruxolitinib (JAK1/JAK2 inhibitor) showed positive results in adults with secondary HLH [27]. Considering involvement of various cytokines in MAS, we agree that Janus family kinase (JAK) inhibitors are emerging as a promising treatment strategy [27, 28].

Half of the patients had an infection during treatment; however, combined infections were controlled in most patients even with corticosteroid and additional immunosuppressant treatment. Four of the five patients who were treated with etoposide experienced fungal and bacterial infection. The immunosuppressive treatment for MAS is a fine balance between the control of inflammation and the prevention of opportunistic infection. However, in the case of MAS, the escape from a vicious cycle of autoreactive inflammation is most important; thus, immunosuppressants should be maintained concurrently with the management of opportunistic infection, even if the doses require to be adjusted.

The present study has several limitations. First, we cannot exclude the selection bias resulting from the retrospective and single-center study design. Second, the number of patients with MAS was insufficient; thus, the study may be underpowered to draw a significant conclusion. Third, although we have tried various drugs to treat MAS, several drugs that are expected to have a therapeutic effect are not administered. The therapeutic effect of anakinra (IL-1 receptor antagonist) has been reported in previous studies, but we could not administer it because it is stored only in orphan drug centers in Korea [1]. Despite these limitations, we identified patients with MAS with rheumatic disease who satisfied all the current criteria, including the HScore. We also attempted to describe the course of management and to establish the difference in clinical features between survivors and non-survivors with MAS.

In conclusions, despite advances in treatment, the mortality of MAS in patients with rheumatic disease remains poor. Renal impairment and low platelet count were associated with poor outcome, while a high level of tissue turnover markers (AST, LDH) was associated with a favorable outcome. Because MAS requires strong immunosuppressive treatment, intensive surveillance for infection is necessary during treatment for MAS.

## Supporting information

S1 File (XLSX)

## References

[1] Crayne CB, Albeituni S, Nichols KE, Cron RQ. The immunology of macrophage activation syndrome. Front Immunol. 2019; 10: 119. doi: 10.3389/fimmu.2019.00119 30774631 PMC6367262

[2] Carter SJ, Tattersall RS, Ramanan AV. Macrophage activation syndrome in adults: recent advances in pathophysiology, diagnosis and treatment. Rheumatology (Oxford). 2019; 58: 5–17. doi: 10.1093/rheumatology/key006 29481673

[3] Lerkvaleekul B, Vilaiyuk S. Macrophage activation syndrome: early diagnosis is key. Open Access Rheumatol. 2018; 10: 117–128. doi: 10.2147/OARRR.S151013 30214327 PMC6124446

[4] Deane S, Selmi C, Teuber SS, Gershwin ME. Macrophage activation syndrome in autoimmune disease. Int Arch Allergy Immunol. 2010; 153: 109–120. doi: 10.1159/000312628 20407267

[5] La Rosee P, Horne A, Hines M, von Bahr Greenwood T, Machowicz R, Berliner N, et al. Recommendations for the management of hemophagocytic lymphohistiocytosis in adults. Blood. 2019; 133: 2465–2477. doi: 10.1182/blood.2018894618 30992265

[6] Otrock ZK, Eby CS. Clinical characteristics, prognostic factors, and outcomes of adult patients with hemophagocytic lymphohistiocytosis. Am J Hematol. 2015; 90: 220–224. doi: 10.1002/ajh.23911 25469675

[7] Zhao Y, Lu D, Ma S, Li L, Zhu J, Zhou, et al. Risk factors of early death in adult patients with secondary hemophagocytic lymphohistiocytosis: a single-institution study of 171 Chinese patients. Hematology Am Soc Hematol Educ Program. 2019; 24: 606–612. doi: 10.1080/16078454.2019.1660458 31474196

[8] Birndt S, Schenk T, Heinevetter B, Brunkhorst FM, Maschmeyer G, Rothmann F, et al. Hemophagocytic lymphohistiocytosis in adults: collaborative analysis of 137 cases of a nationwide German registry. J Cancer Res Clin Oncol. 2020; 146: 1065–1077. doi: 10.1007/s00432-020-03139-4 32076823 PMC7085479

[9] Barba T, Maucort-Boulch D, Iwaz J, Bohe J, Ninet J, Hot A, et al. Hemophagocytic lymphohistiocytosis in intensive care unit: a 71-case strobe-compliant retrospective study. Medicine (Baltimore). 2015; 94: e2318. doi: 10.1097/MD.0000000000002318 26705219 PMC4697985

[10] Fukaya S, Yasuda S, Hashimoto T, Oku K, Kataoka H, Horita T, et al. Clinical features of haemophagocytic syndrome in patients with systemic autoimmune diseases: analysis of 30 cases. Rheumatology (Oxford). 2008; 47: 1686–1691. doi: 10.1093/rheumatology/ken342 18782855

[11] Kumakura S, Murakawa Y. Clinical characteristics and treatment outcomes of autoimmune-associated hemophagocytic syndrome in adults. Arthritis Rheumatol. 2014; 66: 2297–2307. doi: 10.1002/art.38672 24756912 PMC4271677

[12] Gavand PE, Serio I, Arnaud L, Costedoat-Chalumeau N, Carvelli J, Dossier A, et al. Clinical spectrum and therapeutic management of systemic lupus erythematosus-associated macrophage activation syndrome: a study of 103 episodes in 89 adult patients. Autoimmun Rev. 2017; 16: 743–749. doi: 10.1016/j.autrev.2017.05.010 28483541

[13] Naveen R, Jain A, Muhammed H, Gupta L, Misra DP, Lawrence A, et al. Macrophage activation syndrome in systemic lupus erythematosus and systemic-onset juvenile idiopathic arthritis: a retrospective study of similarities and dissimilarities. Rheumatol Int. 2021; 41: 625–631. doi: 10.1007/s00296-020-04763-6 33388903

[14] Dhote R, Simon J, Papo T, Detournay B, Sailler L, Andre MH, et al. Reactive hemophagocytic syndrome in adult systemic disease: report of twenty-six cases and literature review. Arthritis Rheum. 2003; 49: 633–639. doi: 10.1002/art.11368 14558048

[15] Shabbir M, Lucas J, Lazarchick J, Shirai K. Secondary hemophagocytic syndrome in adults: a case series of 18 patients in a single institution and a review of literature. Hematol Oncol. 2011; 29: 100–106. doi: 10.1002/hon.960 20809477

[16] Ruscitti P, Cipriani P, Ciccia F, Masedu F, Liakouli V, Carubbi F, et al. Prognostic factors of macrophage activation syndrome, at the time of diagnosis, in adult patients affected by autoimmune disease: analysis of 41 cases collected in 2 rheumatologic centers. Autoimmun Rev. 2017; 16: 16–21. doi: 10.1016/j.autrev.2016.09.016 27664384

[17] Petri M, Orbai AM, Alarcon GS, Gordon C, Merrill JT, Fortin PR, et al. Derivation and validation of the Systemic lupus international collaborating clinics classification criteria for systemic lupus erythematosus. Arthritis Rheum. 2012; 64: 2677–2686. doi: 10.1002/art.34473 22553077 PMC3409311

[18] Yamaguchi M, Ohta A, Tsunematsu T, Kasukawa R, Mizushima Y, Kashiwagi H, et al. Preliminary criteria for classification of adult stills disease. J Rheumatol. 1992; 19: 424–430. WOS:A1992HM91500018 1578458

[19] Henter JI, Horne A, Arico M, Egeler RM, Filipovich AH, Imashuku S, et al. HLH-2004: diagnostic and therapeutic guidelines for hemophagocytic lymphohistiocytosis. Pediatr Blood Cancer. 2007; 48: 124–131. doi: 10.1002/pbc.21039 16937360

[20] Fardet L, Galicier L, Lambotte O, Marzac C, Aumont C, Chahwan D, et al. Development and validation of the HScore, a score for the diagnosis of reactive hemophagocytic syndrome. Arthritis Rheumatol. 2014; 66: 2613–2620. doi: 10.1002/art.38690 24782338

[21] Ravelli A, Minoia F, Davi S, Horne A, Bovis F, Pistorio A, et al. 2016 classification criteria for macrophage activation syndrome complicating systemic juvenile idiopathic arthritis: a European league against rheumatism/American college of rheumatology/paediatric rheumatology international trials organisation collaborative initiative. Ann Rheum Dis. 2016; 75: 481–489. doi: 10.1136/annrheumdis-2015-208982 26865703

[22] Parodi A, Davi S, Pringe AB, Pistorio A, Ruperto N, Magni-Manzoni S, et al. Macrophage activation syndrome in juvenile systemic lupus erythematosus: a multinational multicenter study of thirty-eight patients. Arthritis Rheum. 2009; 60: 3388–3399. doi: 10.1002/art.24883 19877067

[23] Pouchot J, Sampalis JS, Beaudet F, Carette S, Decary F, Salusinsky-Sternbach M, et al. Adult Still’s disease: manifestations, disease course, and outcome in 62 patients. Medicine (Baltimore). 1991; 70: 118–136. 2005777

[24] Gladman DD, Ibanez D, Urowitz MB. Systemic lupus erythematosus disease activity index 2000. J Rheumatol. 2002; 29: 288–291. 11838846

[25] Ahn SS, Yoo BW, Jung SM, Lee SW, Park YB, Song JJ. In-hospital mortality in febrile lupus patients based on 2016 EULAR/ACR/PRINTO classification criteria for macrophage activation syndrome. Semin Arthritis Rheum. 2017; 47: 216–221. doi: 10.1016/j.semarthrit.2017.02.002 28268026

[26] Gualdoni GA, Hofmann GA, Wohlfarth P, Winkler HM, Winkler S, Haslacher H, et al. Prevalence and outcome of secondary hemophagocytic lymphohistiocytosis among SIRS patients: results from a prospective cohort study. J Clin Med. 2019; 8: 541. doi: 10.3390/jcm8040541 31010216 PMC6518152

[27] Ahmed A, Merrill SA, Alsawah F, Bockenstedt P, Campagnaro E, Devata S, et al. Ruxolitinib in adult patients with secondary haemophagocytic lymphohistiocytosis: an open-label, single-centre, pilot trial. Lancet Haematol. 2019; 6: e630–e637. doi: 10.1016/S2352-3026(19)30156-5 31537486 PMC8054981

[28] Wilcox RA. Janus family kinase (JAK) inhibitors in HLH and severe COVID-19. Am J Hematol. 2020; 95: 1448–1451. doi: 10.1002/ajh.25985 32918336

